# Considerations on surgery invasiveness and response and toxicity patterns in classic palliative radiotherapy for acrometastases of the hand: a hint for a potential role of stereotactic body radiation therapy? A case report and literature review

**DOI:** 10.3389/fonc.2023.1146041

**Published:** 2023-06-21

**Authors:** Gianluca Ferini, Valentina Zagardo, Anna Viola, Marco Maria Aiello, Mandara Muralidhar Harikar, Tejas Venkataram, Paolo Palmisciano, Salvatore Ivan Illari, Vito Valenti, Giuseppe Emmanuele Umana

**Affiliations:** ^1^Department of Radiation Oncology, REM Radioterapia Srl, Viagrande, Italy; ^2^Department of Radiation Oncology, Fondazione Istituto Oncologico del Mediterraneo (IOM), Viagrande, Italy; ^3^Medical Oncology, University Hospital Policlinico San Marco, Catania, Italy; ^4^Department of Neurosurgery, Trauma Center, Gamma Knife Center, Cannizzaro Hospital, Catania, Italy; ^5^Department of Neurosurgery, College of Medicine, University of Cincinnati, Cincinnati, OH, United States

**Keywords:** acrometastases, hand metastases, stereotactic body radiotherapy (SBRT), metastatic lung adenocarcinoma, palliative radiation therapy

## Abstract

**Background:**

The rarity of hand acrometastases hampers the consensus-building for their optimal management among the involved oncology professionals. In the current literature, demolitive surgery overcomes the use of palliative radiotherapy, which proved to be ineffective in more than 30% of cases treated with classic palliative dose schemes, carrying also a not negligible radiation-related adverse event rate. Against this background, stereotactic body radiation therapy (SBRT) could emerge as a well-balanced therapeutic option.

**Case summary:**

Here we describe the methods and outcomes of a SBRT treatment of a painful and function-limiting hand acrometastasis in a patient with a history of stage IIIB lung adenocarcinoma. We delivered a total dose of 30 Gy in five daily fractions to a soft-tissue metastasis abutting the fifth metacarpal bone through the SBRT protocol generally used for intracranial treatments. A few weeks later, the patient reported a clinical complete response with acrometastasis and pain disappearance, function recovery, and no significant toxicity. The acrometastasis was the first sign of an atypical cancer progression.

**Conclusions:**

SBRT for hand acrometastases is feasible and might have the best therapeutic profile among the currently available treatment options for this rare clinical scenario. Larger investigations are needed to confirm the present single-case experience.

## Introduction

Acrometastases, namely metastases located distally to the elbow and knee, represent a rare clinical scenario generally indicating a high overall tumor burden and consequently a dismal prognosis, which does not exceed six months of median survival (1). Due to their rarity and absence of pathognomonic signs, acrometastases are often misdiagnosed and confused with benign diseases (i.e. pyogenic granuloma, osteomyelitis, tuberculosis, inflammatory processes, gout, etc.), especially when presenting in the context of undiagnosed cancer (2). The most prevalent primary tumors are lung, kidney, and breast carcinoma. Acrometastases to the hands arise as hot, reddened, growing, and painful nodules swelling the soft tissues and/or destroying the bones (3). This peculiar tumor location may severely impair function and alter quality of life, thus calling for timely diagnosis and treatment. As regards the latter, surgery procedures ranging from curettage to amputation are the most used therapeutic options for local removal (4), relegating radiotherapy (RT) to mere pain control by the classic palliative radiation doses commonly employed in metastatic bone disease (i.e. 8 Gy in single fraction, 20 Gy in five daily fractions, 30 Gy in ten daily fractions) (5). However, such a dose range may be inadequate to effectively shrink any relatively large lesion that disables motor function and causes severe pain due to the over-stretching of the hand’s densely innervated soft tissues (6–9). On the other side, surgery may physically eliminate the source of pain but often at the cost of crippling consequences on hand function (10). Therefore, within a non-standardized therapeutic algorithm for this rare clinical condition, stereotactic body radiation therapy (SBRT) using ablative doses could be introduced to achieve both symptom relief and function recovery, avoiding demolitive surgical procedures. SBRT uses large doses per fraction precisely delivered with specific stereotactic equipment to limit any damage to the healthy tissues surrounding the tumor target (11–14).

Here we describe the methods used for a metastatic lung cancer patient with an acrometastasis to the left hand treated with SBRT, together with the subsequent atypical clinical course.

## Case report

### Clinical presentation

A 57-year-old female with a history of stage IIIB lung adenocarcinoma (biopsy on 12 April 2022: KRAS-G12X, EGFR-wt, BRAF-wt, PDL1-, ALK-, ROS1-, RET-, MET- negative) submitted to concomitant chemo-radiotherapy (56 Gy in standard fractionated involved-field radiotherapy plus cisplatin-pemetrexed) in May-June 2022 needed a 30 Gy-palliative RT course to the right sacroiliac joint for the appearance of a painful gait-limiting metastasis to the corresponding sacral ala the following August, as detected by an 18F-FDG Positron Emission Tomography (PET). Almost simultaneously, the patient started complaining of moderate pain in her left hand, which rapidly developed allodynia, redness, and swelling of the hypothenar eminence and corresponding dorsal side. This greatly limited the hand function. Unfortunately, the PET scan was extended up to the proximal third of the forearms, having been performed with the arms overhead. An ultrasound exam revealed a nodular lesion measuring 3,8x3,1 cm at the palmo-lateral edge of the left hand and no other suspicious findings in the PET-unscanned forearm. A hand X-ray did not detect any macroscopic lysis of the abutting fifth metacarpal bone. The final magnetic resonance imaging (MRI) confirmed the suspicion of a soft tissue acrometastasis closely adherent to the cortical bone of the basis of the fifth metacarpal bone. The patient refused any surgical procedures and was admitted to radiotherapy care. Given the low overall tumor burden (still classifiable as oligoprogressive disease), the patient’s good performance status (Karnofsky score = 80), and our concerns about classic palliative radiotherapy, we decided on SBRT before starting immunotherapy.

### Intervention description

**Figure 1:**
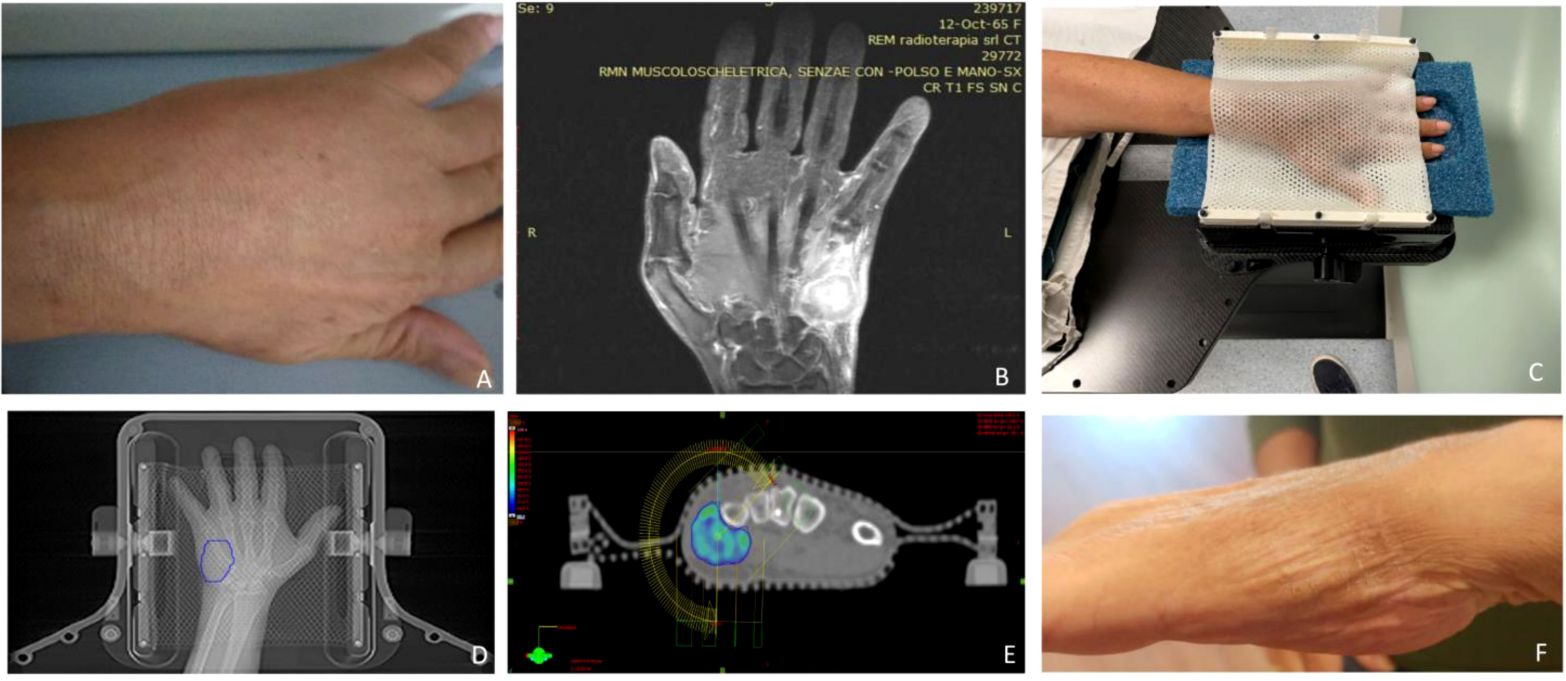
**(A)** Clinical presentation of the acrometastasis; **(B)** MR exam of the left hand; **(C)** set-up procedure; **(D)** contouring process on the CT-simulation; **(E)** 95% dose coverage with the two treatment coplanar arcs highlighted; **(F)** hand status one month after SBRT.

**Figure 2:**
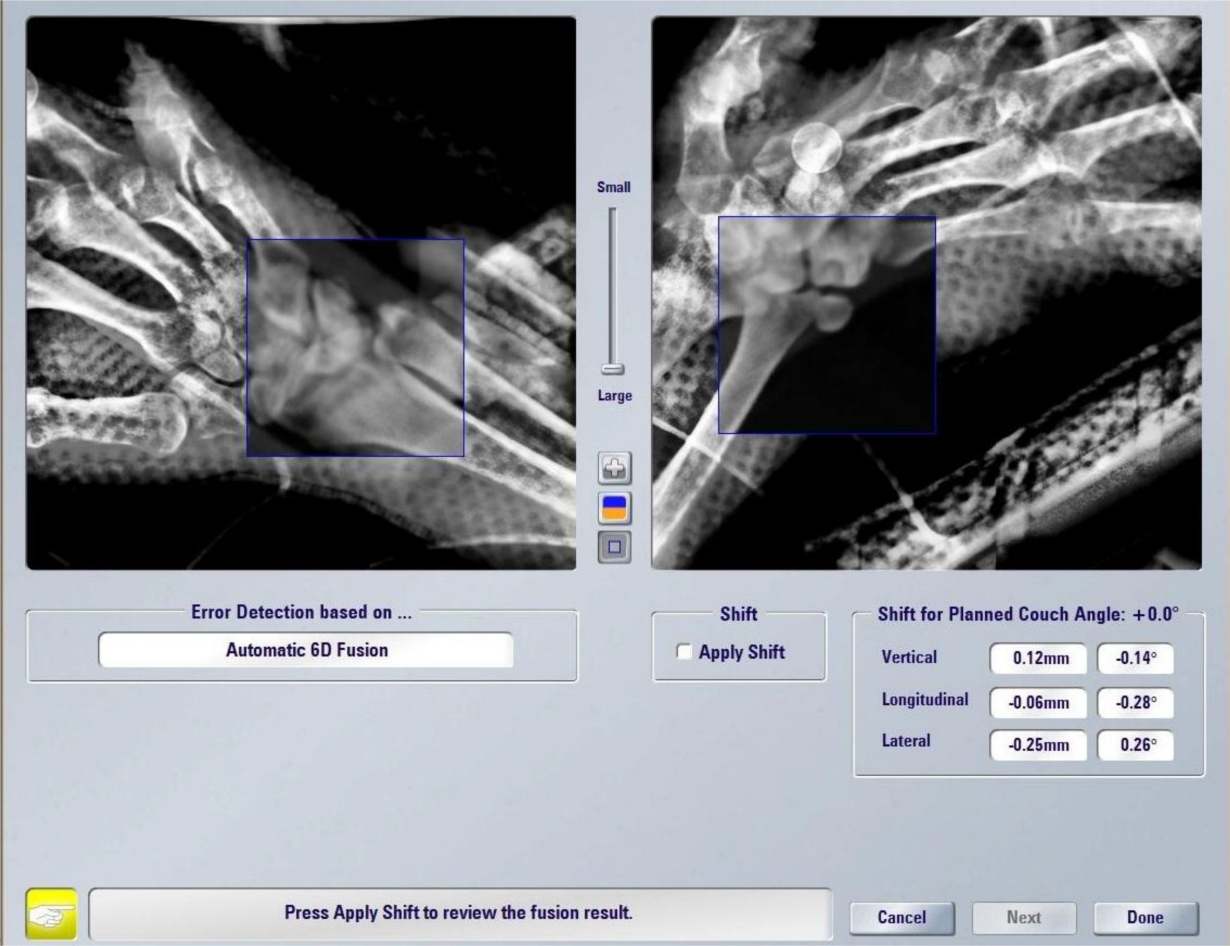
Treatment setup verification by the ExacTrac system.

A prerequisite for high-precision SBRT is a robust immobilization system to ensure a reproducible and accurate setup as much as possible. As we chose to deliver the treatment by a Novalis TrueBeam STx (Varian, Palo Alto, CA, USA), we used the stereotactic equipment for intracranial targets: the patient was positioned prone on the simul-Computed Tomography (CT) couch with her left hand above her head and between a two-sheet stereotactic mask within the frameless extension for ExacTrac™ system (Brainlab^®^, Munich, Germany) at the couch top, the hand replacing the skull (**Figure 1**). The simulation CT scan was acquired with 1.5mm thickness slices and merged with the previous diagnostic MRI for precise delineation of the gross tumor volume (GTV). This was 1 mm-expanded to create a clinical target volume (CTV) covering any possible subclinical spread, especially in the fifth metacarpal bone. A final expansion of 1 mm was added to the CTV for the definition of the planning target volume (PTV) to avoid missing the target for submillimeter displacements, i.e. the largest possible per protocol. We planned a total dose of 30 Gy in five daily fractions and reported 95% of the dose prescription to the 98% of the PTV (with a 108,4% maximum point dose) by two coplanar volumetric modulated arcs (VMAT) (50°-181°CCW/CW). The treatment was delivered under both the ExacTrac- and cone beam CT-guidance in five consecutive days in September 2022 (**Figure 2**).

### Outcomes and follow-up

**Figure 3:**
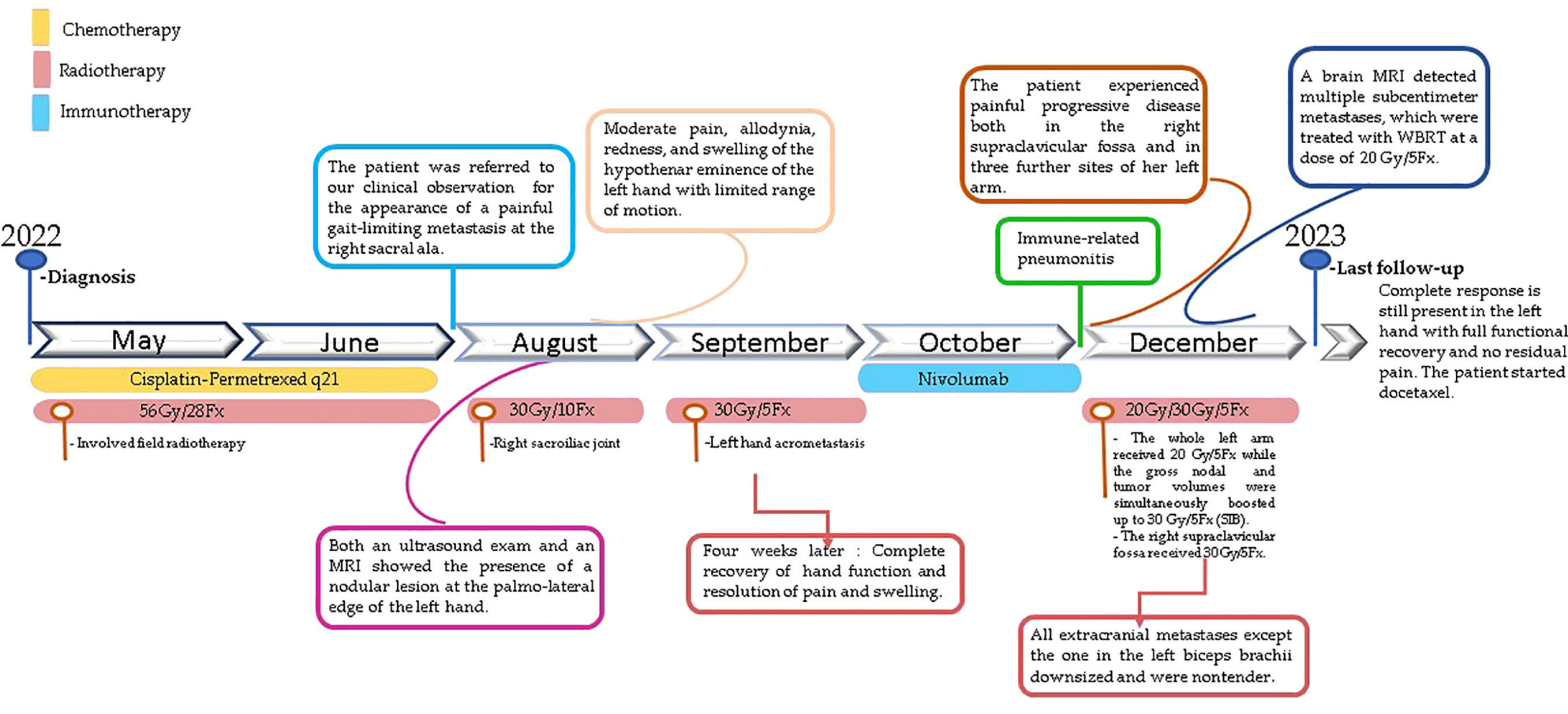
Timeline of events.

The patient experienced a rapid acrometastasis shrinkage with progressive resolution of pain and recovery of hand function, which were complete four weeks after the end of RT. No significant toxicity was complained. Then, the patient started a systemic treatment with Nivolumab, which was suspended just after two cycles due to the occurrence of severe pneumonitis first and of pseudomembranous colitis later, requiring hospitalization for more than one month. Once these intercurrent events passed and before starting any further systemic treatments, the patient returned to our attention due to the painful progression of her metastatic disease at the right supraclavicular fossa (7,8 cm diameter) and at three further sites of her left arm: a large epitrochlear lymph node metastasis (2,7 cm diameter), a soft tissue metastasis inside the bicep muscle (5,5 cm diameter), and bulky matted axillary node metastases (7,7 cm diameter) causing mild arm swelling. In our opinion, the centripetal in transit-like progression of the tumor disease at this stage needed a more inclusive RT approach rather than selectively irradiating only the three metastatic sites of the left arm. Thus, in December 2022, we treated the whole left arm from the shoulder to the elbow with a dose of 20 Gy in five fractions of 4 Gy each while simultaneously boosting (simultaneous integrated boost, SIB) the gross nodal and tumor volumes to 30 Gy (6 Gy/day). The right bulky supraclavicular metastasis was concomitantly treated with a dose of 30 Gy. Just after the completion of these treatments (14 December), the patient performed a brain MRI exam to establish the brain tumor burden as the previous contrast-enhanced CT had detected two small brain metastases, eventually amenable to stereotactic radiosurgery. Unfortunately, the brain MRI revealed multiple disseminated subcentimeter metastases, for which the most appropriate treatment was deemed to be whole-brain radiotherapy. This was delivered with a dose of 20 Gy in five consecutive days to initiate a systemic treatment as early as possible. The patient started docetaxel on 2 January 2023. At the last follow-up (16 January 2023), all four extracranial metastases except the one in the biceps downsized and were nontender while the left hand still presented a sustained complete response with full functional recovery and no residual pain. The brain MRI re-evaluation is awaited in due course (**Figure 3**).

## Discussion

To our knowledge, this is the first case of acrometastasis to the hand treated with SBRT. According to the recent systematic review by Umana et al. (2), surgery is the most common treatment for this metastatic bone site, especially in the form of digital or ray amputation (15). Obviously, such a demolitive surgery has an adverse aesthetic and functional impact and represents a physical and psychological trauma worsening the patient’s daily living (10).

Radiotherapy is a historical therapeutic option for the palliative treatment of painful or complicated bone metastases (5), being able to be also integrated with new systemic anticancer therapies (16). With advancing technology and awareness of the oligometastatic or oligoprogressive status, which deserve dose-escalation aimed at better local control (17, 18), the SBRT use for bone metastases is enormously increased in recent years, being currently tested also for polymetastatic setting (19). Compared to classic palliative RT using low biologically effective doses, SBRT adopts larger doses, which fall steeply off the tumor target, drastically reducing the risk of damage to surrounding healthy tissues exposed to radiation (20–22). In metastatic bone disease, this technique demonstrated to improve the complete pain response rate while reducing the local progression one (23).

Reviewing the literature on hand acrometastases shows that the response to classic palliative RT doses is variable. For example, the clinical results achieved through 30 Gy in 10 fractions ranged from good symptom palliation in three cases (two metacarpal, one phalangeal), modest pain control in another report with metastatic involvement of a distal phalanx, and to no significant benefit with even worsening of swelling in another patient with bilateral carpal bone metastases (6, 24–27). In another case of thumb metastasis, a 30 Gy dose produced a symptomatic improvement, along with soft tissue edema as a collateral event (28). Longer dose fractionation schemes (54 Gy in 27 fractions and 40 Gy in 20 fractions) were equally effective, but more treatment sessions could potentially discomfort patients in terms of compliance with the RT schedule (27, 29). A total dose of 27 Gy, whose fractionation scheme was omitted, resulted in the complete disappearance of both pain and swelling in a patient with a metacarpal bone metastasis (30). Pain and motion complaints resolved after 8 Gy-single fraction administration in other two cases of metacarpal metastases (3). Two further patients benefitted from an 8 Gy-single fraction (31, 32). A dose of 15 Gy in 3 fractions produced only transient pain relief lasting a few weeks (7). Lastly, 20 Gy in 5 fractions produced no response in two patients (8, 9) and complete pain relief and regaining of the full range of motion in another one (33). No indication about the dose used in another mostly ineffective RT treatment for a distal phalanx metastasis was provided by the authors (34). This inconsistency of results motivated us to test an escalation of radiation dose in our case. Indeed, from this pooled cohort, there is evidence of a treatment failure rate and radiation-related adverse event rate equal to 31,6% (6/19) and 10,5% (2/19), respectively. The above findings are summarized in **Table 1**.

**Table 1:** Summary of case reports listing the prescribed radiation dose, fractionation scheme and clinical outcomes.

Authors, Year	Primary tumor histology	Acrometastasis Site	RT Dose/Fractions (Fx)	Outcomes
Khosla et al, 2012 (24)	Vaginal squamous cell carcinoma	Left fourth metacarpal	30Gy/10Fx	Good symptom palliation
De Smet L., 2004 (25)	Breast carcinoma	Right second metacarpal	30Gy/10Fx	Good symptom palliation
Sumodhee et al, 2014 (26)	Bronchogenic adenocarcinoma	Left ring finger	30Gy/10Fx	Good symptom palliation
Kumar et al, 2011 (27)	Cutaneous squamous cell carcinoma	Right tumb, distal phalanx	54Gy/27Fx	Good symptom palliation
	Esophageal squamous cell carcinoma	Left finger, distal phalanx	30Gy/10Fx	Modest pain control
Park et al, 2006 (6)	Gastric adenocarcinoma	Bilateral carpal bones	30Gy/10Fx	Minimal pain control and worsening of swelling
Vasic L., 2010 (28)	Sigmoid colon cancer	Right thumb, proximal and distal phalanges	30Gy/10Fx	Good symptom palliation and onset of soft tissue edema
Tabrizi et al, 2018 (29)	Bronchogenic adenocarcinoma	Left hamate	40Gy/20Fx	Good symptom palliation
Kodama et al, 2009 (30)	Bronchogenic adenocarcinoma	Left metacarpal	27Gy/NR	Complete pain resolution
Flynn et al, 2008 (3)	Bronchogenic non-small cell carcinoma	Left second metacarpal	8Gy/1Fx	Complete pain resolution
	Breast carcinoma	Right third and fifth metacarpal	8Gy/1Fx	Complete pain resolution
Asthana et al, 2001 (31)	Breast carcinoma	Left thumb, proximal phalanx	8Gy/1Fx	Good symptom palliation
Myrehaug and Bezjak, 2010 (32)	Bronchogenic adenocarcinoma	Right index finger, proximal phalanx	8Gy/1Fx	Good symptom palliation
Carvalho H.d.A. et al, 2002 (7)	Bronchogenic small cell carcinoma	Right thumb, distal phalanx	15Gy/3Fx	Transient pain relief
Bigot et al, 2007 (8)	Gastric adenocarcinoma	Right third metacarpal	20Gy/5Fx	No pain control
Verardino et al, 2011 (9)	Basaloid carcinoma of the anal canal	Right and left ring fingers, distal phalanges	20Gy/5Fx	No pain control
Kumar A., 2009 (33)	Gastric adenocarcinoma	Second left metacarpal	20Gy/5Fx	Complete pain resolution
Lambe et al, 2014 (34)	Bronchogenic squamous cell carcinoma	Right fifth finger, distal phalanx	NR	No pain control
Ferini et al, 2023	Lung adenocarcinoma	Soft tissues surrounding the fifth metacarpal	30Gy/5Fx	Complete pain resolution

RT, Radiation Therapy; NR, Not Reported.

Given the excellent performance of easier ultra-hypofractionated RT protocols (without the support of any stereotactic technology) even among frail patients (35, 36), one may question the need for SBRT in the scenario described here. However, it is worth noting that an expanded target volume (i.e. PTV to take into account any positional uncertainties) at this body site may provoke excessive acute skin toxicity, like in Dupuytren’s disease treatment, and a greater risk of bone necrosis (37–39). Moreover, exposing a larger amount of the healthy lymphatic system to harmful radiation can produce chronic lymphedema culminating in function-limiting stiffness of the hand (40–42).

Our SBRT protocol was based on the use of the ExacTrac system, which, despite being specifically designed for intracranial and spine targets (17, 21, 43, 44), proved to be of great accuracy also in the treatment of appendicular skeleton in upper extremities (45). Other than the setup corrections, which notoriously reduce the toxicity rate when done on a daily basis (46), the ExacTrac system allows also intrafraction motion monitoring.

The characteristic tangential effect of rotational RT techniques like that used here reduces the need for a bolus to adequately irradiate even the most superficial layers of the target abutting the skin (47, 48). In agreement with this, we registered that 95% of the dose prescription covered 98,12% of the PTV.

Our patient achieved a rapid complete response in terms of both tumor regression and pain reduction with no significant toxicity, also restoring the full range of motion of her left hand. These findings encourage the introduction of SBRT among the therapeutic options available for the treatment of acrometastases.

The present case report has also the merit to describe an atypical cancer progression along the upper extremity after the appearance of the hand acrometastasis, alerting the practitioners about such a possible disease evolution. Interestingly, the tumor histology of our patient exhibited poor sensitivity to systemic drugs while being a good responder to radiation. This warrants further investigations on the possible cancer cell targets to hit with radionuclide therapeutic agents (49).

## Conclusions

SBRT might be characterized by the most favorable therapeutic profile among the currently available therapeutic options for the treatment of acrometastases. Larger series of patients are needed to confirm the results of our single case description.

## Patient perspective

I am fully satisfied with the clinical result obtained in the hand, confident in achieving the same in the arm, and enormously grateful to the doctors who are supporting me in my fight against cancer.

## Data availability statement

The original contributions presented in the study are included in the article/supplementary material. Further inquiries can be directed to the corresponding author.

## Ethics statement

Ethical review and approval was not required for the study on human participants in accordance with the local legislation and institutional requirements. The patients/participants provided their written informed consent to participate in this study. Written informed consent was obtained from the participant/patient(s) for the publication of this case report.

## Author contributions

GF: writing - original draft. VZ and GF: data collection. AV, MMA, MH, TV, PP: supervision. GF, SI, VV and GU writing - review and editing, also the treating doctors. All authors contributed to the article and approved the submitted version.
